# Use of Free Flaps in the Reconstruction of the Ventral Skull Base: A Systematic Literature Review

**DOI:** 10.1002/hed.70302

**Published:** 2026-05-12

**Authors:** Francesco Mazzola, Francesco Benvegnù, Milena Fior, Martina Conti, Stefano Taboni, Enzo Emanuelli, Philippe Herman, Piero Nicolai, Elisabetta Zanoletti, Raul Pellini, Marco Ferrari

**Affiliations:** ^1^ Unit of Otorhinolaryngology‐Head and Neck Surgery IRCCS Regina Elena Cancer Institute of Rome Rome Italy; ^2^ Section of Otorhinolaryngology‐Head and Neck Surgery, Department of Neurosciences University of Padua Padua Italy; ^3^ Unit of Otorhinolaryngology‐Head and Neck Surgery Ca’ Foncello Hospital, ULSS2 Marca Trevigiana Treviso Italy; ^4^ Otorhinolaryngology and Skull Base Center AP‐HP, Hôpital Lariboisière Paris France

**Keywords:** flap transposition, free flaps, microanastomoses, reconstruction, skull base

## Abstract

**Background:**

Reconstruction of the ventral skull base represents a challenge in head and neck surgery. When local or regional flaps are insufficient, microvascular free flaps offer a reliable reconstructive option.

**Methods:**

A systematic literature review was conducted following PRISMA guidelines to identify studies on ventral skull base reconstruction using microvascular free flaps. Eligible studies included cases with an explicit description of the flap type, recipient vessels, and transposition corridor. The authors propose a categorization of the surgical corridors used for pedicle transposition to optimize result interpretation.

**Results:**

Thirty‐seven studies encompassing 147 patients met inclusion criteria. A possible preferential association between site, corridor, and flap can be suggested.

**Conclusions:**

This study supports the role of a tailored selection of surgical corridors and recipient vessels in optimizing ventral skull base reconstruction. Moreover, it emphasizes the importance of matching anatomical feasibility with flap and patient's characteristics.

## 1 Introduction

The reconstruction of large defects of the ventral skull base (VSB) often represents a surgical challenge, in which the main purpose is to ensure anatomical and functional separation between the intracranial compartment and the sinonasal‐orbital‐nasopharyngeal compartment. Furthermore, reconstruction must provide adequate support for neural tissues, preventing prolapse of brain and meninges, and must guarantee a good healing potential for the nasal mucosal surface. Free flaps are emerging as an interesting relatively novel solution for the reconstruction of extensive skull base defects [1, 2, 3]. The reconstruction site imposes specific selection criteria regarding the type of free flap to be used, due to significant differences in the anatomy of the recipient area, in cerebrospinal fluid (CSF) pressure, and in the impact of the weight of the brain parenchyma on the reconstructed area.

Rarely, it is necessary to use a large amount of vascularized tissue with more reliable vascularity and larger dimensions than widely accepted pedicled flaps [4]. This uncommon clinical situation can be encountered in case of [5]:

- Extensive oncologic resections;
- Severe infections such as osteomyelitis, with ischemia or necrosis of the VSB;
- The need for high‐dose heavy particle radiotherapy as adjuvant treatment;
- Reconstruction on tissue previously treated with radiotherapy;
- Recurrent CSF leak;
- Lack of local and regional reconstructive solutions as a result of previous surgeries.

The use of microvascular free flaps therefore represents a valuable reconstructive option in such situations although, in such a complex scenario, all case‐specific variables must be carefully considered. The available literature on the subject is limited to retrospective case series and case reports from single centers. The objective of the present study is to provide the results of a systematic review of the existing literature on the use of free flaps in skull base reconstruction.

## 2 Materials and Methods

**FIGURE 1:**
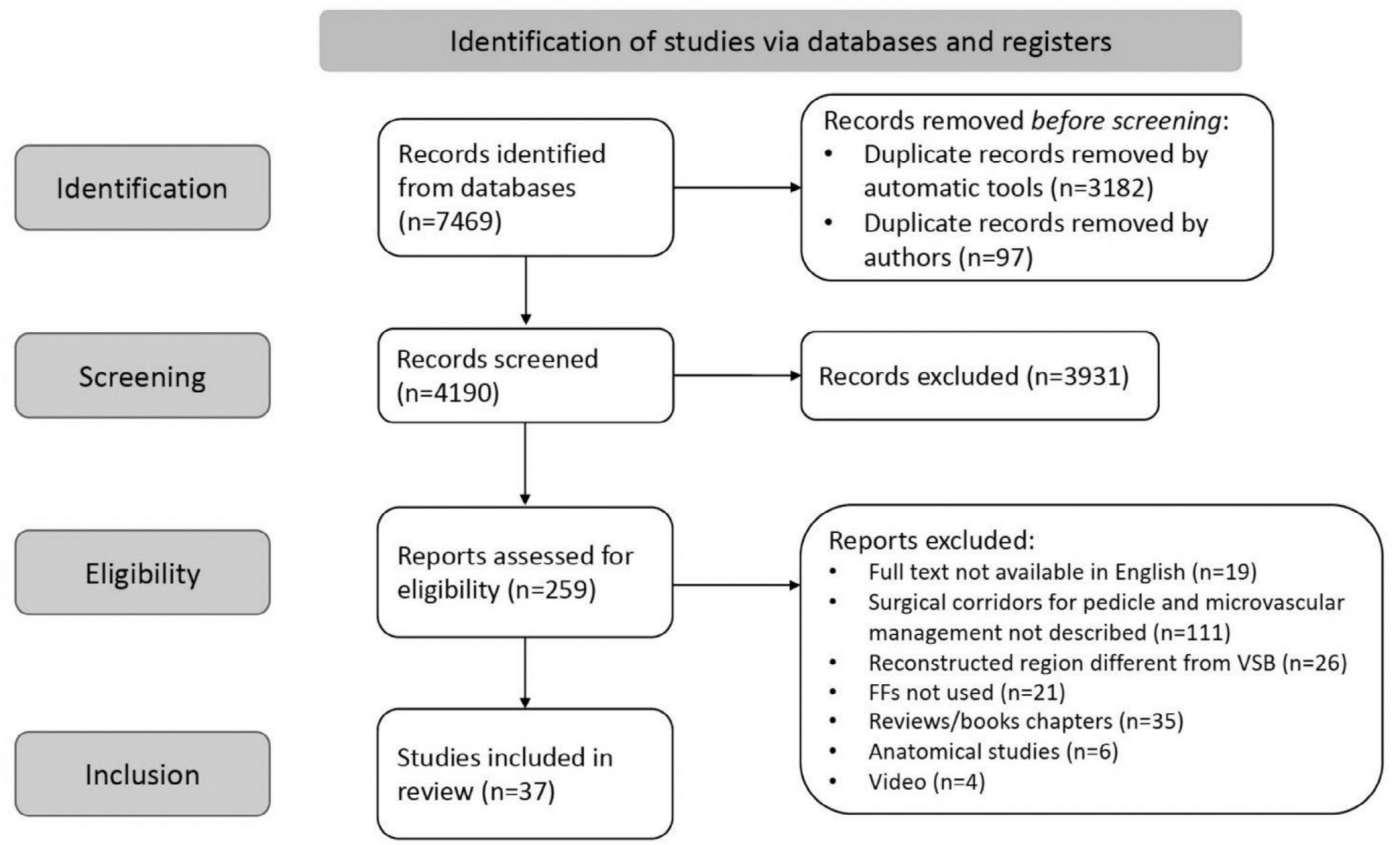
PRISMA flow diagram of literature search and selection process. FFs: Free flaps; VSB: Ventral skull base.

A systematic literature review was conducted according to the Preferred Reporting Items for Systematic Reviews and Meta‐Analyses (PRISMA) recommendations [6]. The electronic databases Scopus, PubMed, Embase and WOS were searched from database inception to January 07th 2026. The inclusion criteria for selection of studies were chosen according to the PICOS tool: Population (P), patients with conditions (oncological and non‐oncological) or anatomical defects of the VSB; Intervention (I), free flap reconstruction; Comparator (C), none; Outcomes (O), free flap vitality, success of the reconstruction; Study design (S), retrospective cohort studies and case series. A combination of MeSH terms and free‐text words was employed. The reference lists of all the articles included were screened to find other relevant publications. References were exported to Zotero bibliography manager (v7.0.27, Center for History and NewMedia, George Mason University, Fairfax, VA, USA). All the results were analyzed, excluding studies not related to the primary outcome of the research. The screening process is summarized in the PRISMA flow diagram provided in Figure 1.

Studies were included only if they simultaneously met the following criteria:

- patients (sample size and demographic data) who underwent reconstruction of the VSB;
- use of microvascular free flaps for surgical reconstruction:
- description of reconstructive strategy details (i.e., type of flap chosen for reconstruction, preparation of the recipient vessels and creation of the ipsilateral surgical corridor for pedicle transposition).

After the full text analysis for each article, the following information was extracted:

- Number of patients included;
- Site of reconstruction (anterior skull base/clivus);
- Surgery technique (endoscopic/open);
- Indication for reconstruction;
- Type of microvascular free flap used;
- Type of corridor used;
- Vessels used for microvascular anastomosis.

Based on anatomical descriptions reported in the literature and on feasibility considerations, a categorization of the surgical corridors used for pedicle transposition has been made by the authors to optimize the interpretation of the results:

- Parapharyngeal–infratemporal corridor (hereafter referred to as “infratemporal”): from the parapharyngeal space to the infratemporal fossa.
- Parapharyngeal corridor: from the parapharyngeal space to the lateral wall of the nasopharynx.
- Retropharyngeal corridor: from the parapharyngeal space to Rosenmüller's fossa or posterior wall of the nasopharynx, dissecting the virtual space between the prevertebral fascia and the superior pharyngeal constrictor muscle.
- Transmaxillary corridor: from the neck level Ib through a corridor running parallel to the buccinator muscle, reaching the anterior maxillary sinus through a Caldwell–Luc anterior maxillectomy.
- Superficial paratemporal corridor: from the pretragal/preauricular area, where superficial temporal vessels (STV) are located, to the orbito‐zygomatic region through a corridor superficial to the superficial musculo‐aponeurotic system (SMAS).
- Deep paratemporal corridor: from the pretragal/preauricular area to the orbit, infratemporal fossa or skull base through a corridor that extends deeply to the superficial fascia of the temporalis muscle.

## 3 Statistical Analysis

For each discrete variable, the frequency was calculated over the total number of patients considered. For each group, a histogram was created to show the relative frequency with respect to the total population. Histograms were generated using Google Sheets (Google LLC).

## 4 Results

The systematic review led to the selection of 37 articles. The data obtained are summarized in Table 1.

**TABLE 1:** Summary of data extraction.

No.	Authors	Corridor	Type of surgery	No. of patients	Reconstruction site	Purpose of reconstruction	Free flap	Vessels for anastomosis
1	Haynes et al. [7]	Transmaxillary	E + O	1	Anterior skull base	Cancer	RFFF	Facial vessels
2	Lo et al. [8]	Deep paratemporal	O	1	Anterior skull base	Cancer	ALT + vastus lateralis	STV
3	Sorrentino et al. [9]	Transmaxillary	O	2	Anterior skull base	Cancer	ALT	Facial vessels
4	Ungar et al. [10]	Deep paratemporal	O	13	Anterior skull base	ORN	9 ALT, 2 VRAM, 2 RFFF	9 STV, 4 facial vessels
5	Chapchay et al. [11]	Parapharyngeal	E	1	Clivus	ORN	ALT	Facial artery, EJV
6	Kim et al. [12]	Deep paratemporal	O	5	Anterior skull base	Cancer	5 ALT	STV
7	Auricchio et al. [13]	Deep paratemporal	O	1	Anterior skull base	Infection	ALT	STV
8	Krishnan et al. [14]	Deep paratemporal	O	1	Anterior skull base	CSF leak	RFFF	STV
9	Krane et al. [15]	Parapharyngeal	E	1	Clivus	Infection	Serratus anterior	Superior thyroid artery, facial vein
10	Lee et al. [16]	Transmaxillary	E	3	Anterior skull base	CSF leak	RFFF	Facial vessels
11	Hackman et al. [17]	Transmaxillary	E	1	Clivus	Cancer + ORN	RFFF	Facial vessels
12	Pipkorn et al. [18]	Transmaxillary	E	6	Anterior skull base	5 CSF leak, 1 ORN	RFFF	Facial vessels
13	Rodriguez‐Lorenzo et al. [19]	Transmaxillary	E	5	Anterior skull base	Cancer	1 ALT, 4 vastus lateralis, 1 RFFF	Facial vessels
14	Pipkorn et al. [20]	Transmaxillary	E	1	Anterior skull base	CSF leak	RFFF	Facial vessels
15	Qadeer et al. [21]	Transmaxillary	E + O	1	Clivus	CSF leak	ALT	Facial vessels
16	Sinha et al. [22]	Transmaxillary	E	5	Anterior skull base	CSF leak	RFFF	Facial vessels
17	Sagheer et al. [23]	2 Retropharyngeal 3 Parapharyngeal, 2 Transmaxillary	E	7	Clivus	Cancer	6 ALT, 1 RFFF	Arteries: 4 facial artery, 2 ECA, 1 angolar artery
18	Sreenath et al. [24]	Parapharyngeal‐parabuccal	E	5	Clivus	ORN	5 ALT	Facial vessels
19	Inman et al. [25]	Deep paratemporal	O	11	Anterior skull base	CSF leak	8 RFFF, 3 VRAM	Arteries: superficial temporal a.; veins: 5 superficial temporal v., 6 retromandibular v.
20	Vuola et al. [26]	Deep paratemporal	O	30	Anterior skull base	Cancer	19 VRAM, 8 latissimus dorsi, 3 RFFF	Superficial temporal artery 60%, facials artery 23%, others 17%
21	Heredero et al. [27]	Deep paratemporal	O	1	Anterior skull base	Cancer	FFF	STV
22	Minchew et al. [28]	Deep paratemporal	O	11	Anterior skull base	6 Cancer, 4 CSF leak, 1 infection	6 RFFF, 5 VRAM	STV
23	Porto et al. [29]	Infratemporal	E	1	Anterior skull base	Cancer	RFFF	Facial vessels
24	Biron et al. [30]	Deep paratemporal	O	3	Anterior skull base	Infection, 2 CSF leak	RFFF	STV
25	Weber et al. [31]	Deep paratemporal	O	11	Anterior skull base	CSF leak	RFFF	4 STV, 7 facial vessels
26	Moy et al. [32]	Transmaxillary	E	1	Clivus	CSF leak	RFFF	Facial vessels
27	Langston et al. [33]	Deep paratemporal	E	1	Anterior skull base	Cancer	RFFF	STV
28	Vieira et al. [34]	Retropharyngeal	E	1	Clivus	CSF leak, ORN	ALT	Facial vessels
29	Emanuels et al. [35]	Deep paratemporal	O	1	Anterior skull base	Cancer	Vastus lateralis	STV
30	Yeo et al. [36]	Deep paratemporal	O	1	Anterior skull base	Cancer	RFFF	STV
31	London et al. [37]	Retropharyngeal	E	1	Clivus	ORN	RFFF	Thyroid artery, facial vein
32	Kokosis et al. [38]	Deep paratemporal	O	1	Anterior skull base	Infection, CSF leak	O‐FAFF	STV
33	Teknos et al. [2]	Deep paratemporal	O	3	Anterior skull base	Cancer	RFFF	Facial vessels
34	Dhanda et al. [39]	Deep paratemporal	O	1	Anterior skull base	ORN	ALT	STV
35	Kang et al. [40]	Transmaxillary	E	4	Anterior skull base	CSF leak	Vastus lateralis	Facial vessels
36	Lorenzini et al. [41]	Deep paratemporal	O	1	Anterior skull base	CSF leak	RFFF	STV
37	Pangrazi et al. [42]	Deep paratemporal	O	3	Anterior skull base	Infection	RFFF	2 STV, 1 facial artery, 1 EJV

Abbreviations: ALT, Anterolateral thigh flap; CSF, Cerebrospinal fluid; E, Endoscopic; ECA, External carotid artery; EJV, External jugular vein; FFF, Fibular free flap; O, Open; O‐FAFF, Omental fat‐augmented free flap; ORN, Osteoradionecrosis; RFFF, Radial forearm free flap; STV, Superficial temporal vessels; VRAM, Vertical rectus abdominis myocutaneous flap.

The reported studies involved a total of 147 patients.

**FIGURE 2:**
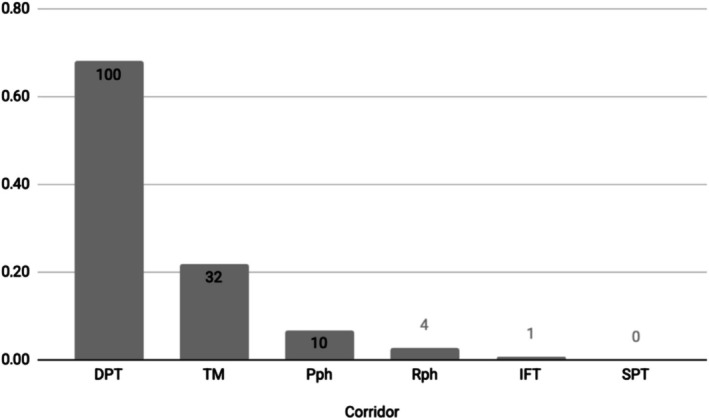
Numerical distribution of all the used corridors among the totality of patients. DPT: Deep paratemporal; IFT: Infratemporal; Pph: Parapharyngeal; Rph: Retropharyngeal; SPT: Superficial paratemporal; TM: Transmaxillary.

The most frequently used corridor according to the analyzed series was the deep paratemporal corridor (100 patients, 68%) (Figure 2). Some of the included studies reported ASB reconstruction performed during extensive craniofacial resections, which in some cases included orbital removal, complete frontal bone or anterior frontal plate ablation, or frontal sinus cranialization. In these cases, the pedicle was often transposed laterally toward the superficial temporal vessels, passing alongside or through the temporalis muscle; for this reason, such cases were included within the category of the deep paratemporal corridor. The second most frequently described corridor was the transmaxillary one (32 patients, 22%), followed by the parapharyngeal corridor (11 patients, 7%) and the retropharyngeal corridor (4 patients, 3%). The infratemporal corridor was described in only 1 study (1%), specifically in a case of ASB reconstruction. In the analyzed series, no use of the superficial paratemporal corridor was reported.

**FIGURE 3:**
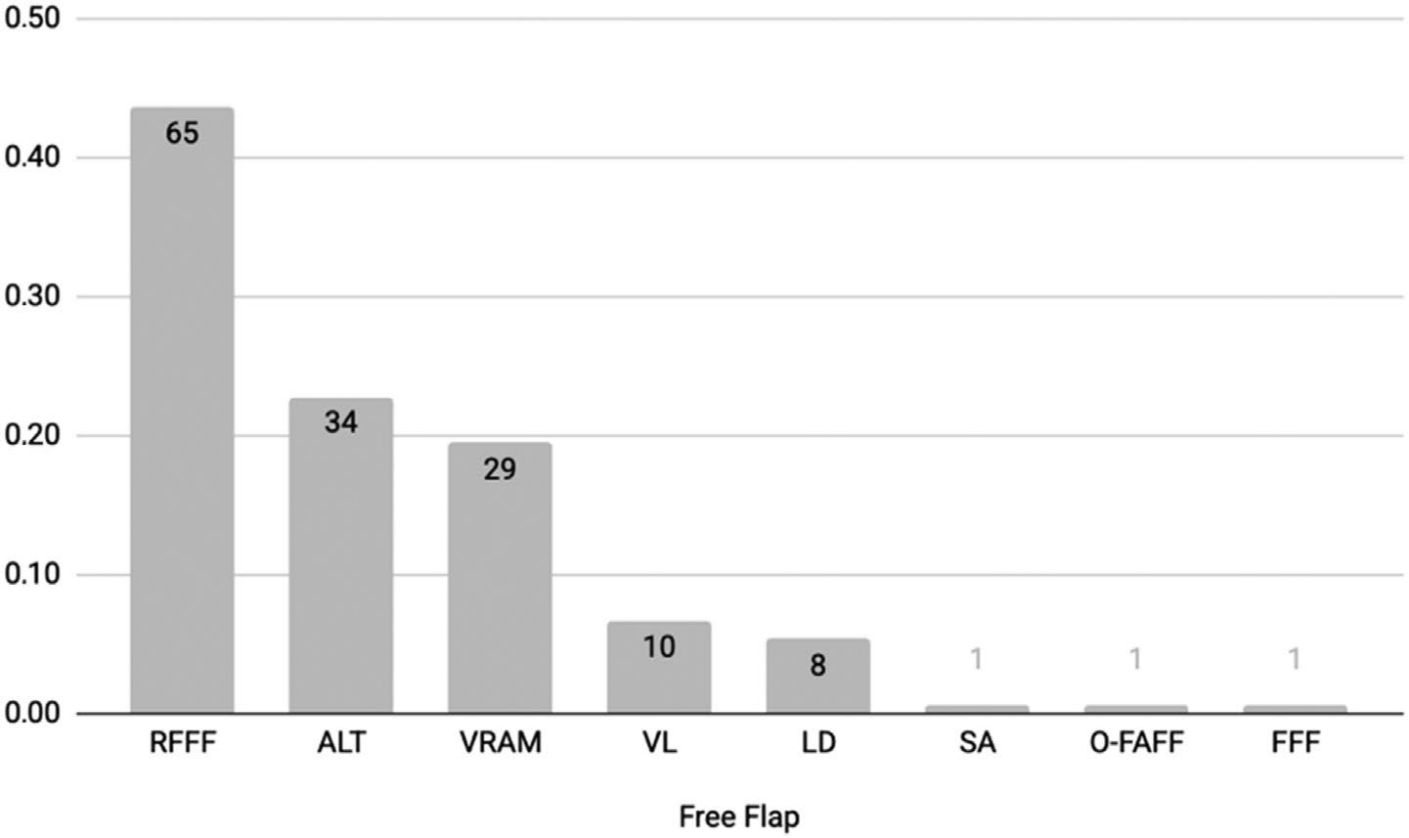
Numerical distribution of all the used free flaps among the totality of patients. ALT: Anterolateral thigh flap; FFF: Fibular free flap; LD: Latissimus dorsi flap; O‐FAFF: Omental fat‐augmented free flap; RFFF: Radial forearm free flap; SA: Serratus anterior flap; VL: Vastus lateralis flap; VRAM: Vertical rectus abdominis myocutaneous flap.

The most frequently used free flap was the radial forearm free flap (RFFF) (68 patients, 43%), followed by the anterolateral thigh flap (ALT) (33 patients, 21%) and the vertical rectus abdominis myocutaneous flap (VRAM) (29 patients, 18%) (Figure 3). Other less commonly used flaps were the serratus anterior flap (SA) (11 patients, 7%), the latissimus dorsi flap (LD) (8 patients, 5%), and the vastus lateralis flap (VL) (6 patients, 4%). The omental fat‐augmented free flap (O‐FAFF) and fibular free flap (FFF) were each reported in a single case (1 patient, 0.6%).

**FIGURE 4:**
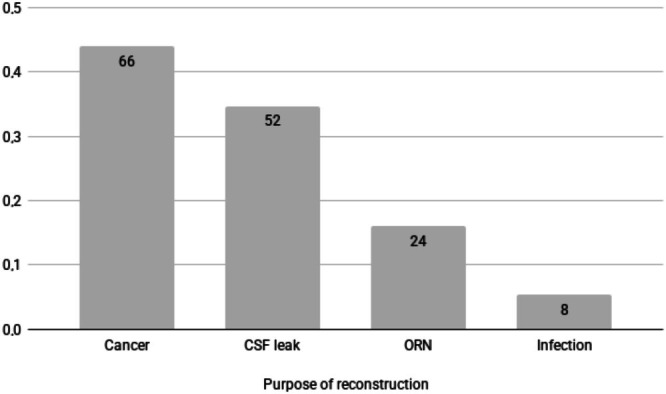
Numerical distribution of the indication for reconstruction among the totality of patients. CSF: Cephalo‐spinal fluid; ORN: Osteoradionecrosis.

Indication for reconstruction involved oncologic patients requiring extensive resections (66 patients, 44%), recurrent CSF (52 patients, 35%), osteoradionecrosis (ORN) (24 patients, 16%), and recurrent infections of the skull base and brain tissues (8 patients, 5%) (Figure 4).

Open surgery was the most used approach (101 patients, 69%), followed by endoscopic techniques (44 patients, 30%). Combined open–endoscopic approaches were used in a small proportion of cases (2 patients, 1%).

The reconstruction involved the VSB, including the posterior wall of the frontal sinus (128 patients, 87%) and the clivus (19 patients, 13%).

The most frequently recipient artery was the superficial temporal artery (73 patients, 50%), followed by the facial artery (64 cases, 44%), the superior thyroid artery (5 cases, 3%), the external carotid artery (2 cases, 1%), other unspecified arteries (2 cases, 1%), and the angular artery (1 case, 0.7%).

The most frequently recipient vein was the facial vein (67 cases, 46%), followed by the superficial temporal vein (66 cases, 45%), the retromandibular vein (6 cases, 4%), the external jugular vein (5 cases, 3%), and other unspecified veins (3 cases, 2%).

## 5 Discussion

### 5.1 Selection of the Surgical Corridor

A clear predominance of the deep paratemporal corridor and STV was observed compared with other options. Several studies highlighted the growing role of the STV as recipient vessels in the reconstruction of the upper and anterior craniofacial areas [43, 44]. The main advantages of using this corridor are its proximity to the reconstructed site, hence requiring a shorter pedicle, and the possibility of achieving reliable anastomoses also in cases of a vessel‐depleted neck. In some cases, authors reported that, to enable passage from the intracranial or frontal space to the temporal region, it was necessary to create a burr hole allowing the pedicle to cross the bony boundary [2]. Pedicle transposition was performed along the plane between the lateral aspect of the skull and the superficial temporalis fascia, while minimizing the risk to the frontal branch of the facial nerve. The corridor was harvested from the pretragal region to the infratemporal fossa, reaching either the posterior wall of the maxillary sinus or the anterior cranial base. The transmaxillary corridor was found to be the second most frequently used. Its main advantages include a minimally invasive preparation, the application for anterior skull base reconstructions and, in some cases, for resurfacing of the nasopharyngeal and clival regions [16, 17]. An element influencing the choice of the flap is the need for a pedicle volumetrically suitable for passage through a maxillotomy of limited dimensions, consisting solely of the vascular pedicle and without additional muscle or adipose tissue. In our series, RFFF, ALT, and vastus lateralis flaps were used for this corridor. These flaps appear to be the most suitable for meeting the mentioned criteria. The parapharyngeal and retropharyngeal corridors were used for the reconstruction of clival defects in all the analyzed series, suggesting an anatomical predisposition for the reconstruction of this region. Both these corridors target the torus tubarius area and differ in their course—respectively anterior and posterior to the internal carotid artery—thus requiring a transcervical dissection time, with a consequent potential increase in surgical morbidity (i.e., intraoperative complications, dysphagia, nerve palsy). The infratemporal corridor is described by Porto et al. [29] as the route of transposition for the pedicle of a de‐epithelialized RFFF, used to seal an VSB defect after the failure of other reconstructive options. Although rarely mentioned, we believe that this corridor should be taken into consideration in order to follow a more physiological anatomical pathways, such as the course of the internal maxillary artery.

### 5.2 Selection of the Flap

In the reconstruction of ventral skull base defects requiring free flaps, the complex three‐dimensional anatomy of the region and the presence of specific bony boundaries make it necessary to select flaps that can be easily shaped to the defect and that have a vascular pedicle of sufficient length to reach suitable recipient vessels for anastomosis. For both these reasons, the RFFF predominates in the analyzed series, as it is well suited for reconstructing most VSB defects. From a technical standpoint, some authors emphasized the importance of harvesting a purely fascio‐adipose RFFF, as this reduces postoperative crusting and cacosmia, facilitates the insetting and minimizes donor‐site morbidity [18]. The ALT represents the second most frequently used flap. In many cases, its use has been described for the reconstruction of clival defects [11, 21, 23, 24, 34], due to its greater volume, which can be shaped to the extent of the defect. This characteristic makes ALT more suitable for larger volumetric defects or when a “sealing” potential is required, such as in case of high pressure CSF leakage [45]. In other cases, the ALT has been utilized to cranialize the frontal or sphenoidal sinuses, with subsequent anastomosis to the superficial temporal vessels [8, 12]. Other described reconstruction techniques involved the use of additional free flaps (i.e., VRAM, SA, LD, VL), selected according to the needs for volumetric and functional filling, the potential for simultaneous two‐team surgery, and the surgeons' preferences and experience [8, 10, 15, 19, 25, 26, 28, 35, 40].

### 5.3 Indications for Reconstruction

In the reviewed literature, oncologic disease represented the most frequent indication for free flap reconstruction. In general, this finding correlates with two primary requirements of oncologic surgical management: (a) obtaining clear safety margins around the pathological tissue, which may necessitate enlargement of the final defect and/or exposure of critical structures (i.e., internal carotid artery, orbital content, meninges, brain); (b) providing a well‐vascularized tissue substrate that minimizes the toxic effects of the adjuvant therapy, when indicated. Thus, Thariat et al. suggest the use of well‐vascularized tissue in anticipation of radiotherapy to separate critical spaces, reduce the risk of fistula formation, and improve healing outcomes [46]. Therefore, while local or regional flaps remain the first‐line reconstructive option for VSB defects, there are situations in which the use of a free flap could be significantly advantageous. Recurrent CSF leaks may benefit from free flap reconstruction to ensure a higher potential for durable healing and watertight sealing. Conversely, in cases of ORN, the reconstructive goal is the revascularization of devitalized, ischemic tissue in order to restore tissue viability and improve clinical outcomes [47]. In a comparable way, in cases of infection, tissue revascularization may facilitate resolution of the infectious process and promote local healing.

### 5.4 Reconstructed Anatomical Region

In our series, the most frequently reconstructed region was the anterior skull base, which, according to the scientific literature, is also the area most affected by the two main indications for reconstructive surgery—namely, cancer and CSF leakage, whether spontaneous, post‐traumatic, or iatrogenic [48]. Based on the findings of the included studies, the definition of anterior skull base resection remains rather unspecific, potentially including involvement of the orbit, frontal sinus, cribriform plate, and planum sphenoidale in varying proportions.

### 5.5 Selection of Vessels for Microanastomosis

The recipient vessels most commonly used for microvascular anastomosis are the STV. Several studies have demonstrated the reliability and versatility of the STV in the reconstruction of head and neck defects [43, 49, 50]. Their main advantages include a short pedicle length required, an easy anatomical accessibility without the need for a cervical surgical phase and their applicability in irradiated or vessel‐depleted neck. During the preparatory phase, careful identification and preservation of both vessels is crucial, as the course of the superficial temporal vein is less predictable. Some authors underline that the small vessel diameter could lead to difficulties during anastomosis and frequent vasospasm, potentially increasing the risk of flap necrosis [51, 52]. The middle temporal vein could be an alternative when the superficial temporal vein is not reliable or too small [51, 53]. The second most frequently selected pair of vessels are the facial vessels, which are typically isolated and transected within the submandibular space or at the mandibular border. The facial vessels represent workhorse recipient vessels in head and neck reconstruction, due to their favorable diameter, ease of dissection from surrounding tissues, good mobility, and their location in a region accessible both from anterior (e.g., transmaxillary) and posterior corridors (e.g., parapharyngeal or retropharyngeal). Iacoviello et al., in selecting the arterial recipient vessel, have proposed a spatial classification based on axial thirds of the head using the superficial temporal artery for defects of the upper third, the facial artery for the middle third, and either the facial or superior thyroid artery for the lower third [54]. However, other factors including the conditions of the recipient vessels as well as the need for matching the recipient vessel diameter to that of the flap pedicle should be taken into consideration. Other vessels used for arterial or venous anastomoses have been reported in case series adopting a patient‐tailored decision‐making approach [11, 15, 23, 25, 26, 37, 42]. In these cases the choice was determined by the residual vascularization after the cervical phase, the condition of the tissues following prior treatments, and the surgeon's specific preferences.

### 5.6 Limits of the Study

The main limitations of our study are the relatively small sample size and treatment heterogeneity, reflecting the low frequency of skull base reconstruction with free flaps and the resulting difficulty in standardizing surgical approaches. Most included studies are small case series and retrospective reports with highly heterogeneous reporting of outcomes and complications. Consequently, a formal risk of bias assessment would likely reflect the inherent limitations of this literature rather than provide meaningful comparative insight. In addition, many studies did not consistently report data on prior surgical procedures, previous reconstruction attempts, or outcomes such as CSF leak closure and flap survival. Extracting these variables would therefore describe only a limited subset of an already highly selected population undergoing free‐flap reconstruction of the ventral skull base. Overall, greater standardization of surgical indications and more homogeneous reporting of techniques and results could enable a more comprehensive analysis of the reconstructive approach under investigation.

## 6 Conclusions

The description and use of different surgical corridors for the transposition of free flap pedicles used in VSB reconstruction address the inherent variability of a procedure that remains patient‐tailored. The study suggests an individualized approach to the reconstruction of the VSB: for the most frequently reconstructed sites, which are located in the anterior skull base, the deep paratemporal and transmaxillary corridors are more often resorted to, while the retro‐ and parapharyngeal corridors are particularly suitable for the clival area. Matching anatomical feasibility with flap characteristics enables tailored surgical planning, improving reconstructive outcomes and reducing procedure‐related morbidity. Standardization of indications and transposition routes could represent a path to improve preoperative planning and the overall management of the patients.

## Funding

The authors have nothing to report.

## Disclosure

Francesco Mazzola is the recipient of the Tor Vergata PhD program in Tissue Engineering and Remodeling Biotechnologies for Body Function.

## Conflicts of Interest

The authors declare no conflicts of interest.

## Data Availability

Data sharing is not applicable to this article as no new data were created or analyzed in this study. All data analyzed during this study are included in the published articles cited in the reference list. The data set used to support the findings of this study (extracted from the included original articles) is available from the corresponding author upon reasonable request.
